# Correction to: Spatial patterns in phage-*Rhizobium* coevolutionary interactions across regions of common bean domestication

**DOI:** 10.1038/s41396-021-00963-5

**Published:** 2021-04-14

**Authors:** Jannick Van Cauwenberghe, Rosa I. Santamaría, Patricia Bustos, Soledad Juárez, Maria Antonella Ducci, Trinidad Figueroa Fleming, Angela Virginia Etcheverry, Víctor González

**Affiliations:** 1 Centro de Ciencias Genómicas, Universidad Nacional Autonóma de México, Mexico, Mexico; 2 Department of Integrative Biology, University of California, Berkeley, CA USA; 3 Instituto Nacional de Tecnología Agropecuaria, Universidad Nacional de Salta, Salta, Argentina; 4 Facultad de Ciencias Naturales, Universidad Nacional de Salta, Salta, Argentina

**Keywords:** Microbial ecology, Bacteriophages, Microbial ecology, Biogeography, Microbial communities

Correction to: *The ISME Journal*

10.1038/s41396-021-00907-z

The article “Spatial patterns in phage-Rhizobium coevolutionary interactions across regions of common bean domestication”, written by Jannick Van Cauwenberghe, Rosa I. Santamaría, Patricia Bustos, Soledad Juárez, Maria Antonella Ducci, Trinidad Figueroa Fleming, Angela Virginia Etcheverry and Víctor González, was originally published electronically on the publisher’s internet portal on 08 February 2021 without open access. With the author(s)’ decision to opt for Open Choice the copyright of the article changed on 28 February 2021 to © The Author(s) 2021 and the article is forthwith distributed under a Creative Commons Attribution 4.0 International License, which permits use, sharing, adaptation, distribution and reproduction in any medium or format, as long as you give appropriate credit to the original author(s) and the source, provide a link to the Creative Commons licence, and indicate if changes were made. The images or other third party material in this article are included in the article’s Creative Commons licence, unless indicated otherwise in a credit line to the material. If material is not included in the article’s Creative Commons licence and your intended use is not permitted by statutory regulation or exceeds the permitted use, you will need to obtain permission directly from the copyright holder. To view a copy of this licence, visit http://creativecommons.org/licenses/by/4.0/.

