# Supplementary material for: Spatial patterns in phage-Rhizobium coevolutionary interactions across regions of common bean domestication
Source: ISME J. 2021 Feb 8;15(7):2092–106. doi: 10.1038/s41396-021-00907-z (PMC8245606; doi:10.1038/s41396-021-00907-z)
Supplement: Supplementary file 1 — Supplemental material [file 41396_2021_907_MOESM1_ESM.pdf]

Supplementary material.

Spatial patterns in phage-*Rhizobium* coevolutionary interactions across regions of common bean domestication

Running title: Spatial patterns in phage-*Rhizobium* interactions.

Jannick Van Cauwenberghe [1,2 *], Rosa I. Santamaría [1, *], Patricia Bustos [1], Soledad Juárez [1], Maria Antonella Ducci[3], Trinidad Figueroa Fleming[4], Angela Virginia Etcheverry[4], and Víctor González [1].

[1] Centro de Ciencias Genómicas, Universidad Nacional Autonóma de México, Mexico**.**

[2] Department of Integrative Biology, University of California, Berkeley, CA, USA

[3] Instituto Nacional de Tecnología Agropecuaria, Universidad Nacional de Salta, Argentina.

[4] Facultad de Ciencias Naturales, Universidad Nacional de Salta, Salta, Argentina.

Supplementary method S1.

Obtention of *Rhizobium* strains from nodules.

Before inoculation with soil, bean seeds were surface sterilized in 70% ethanol for 1 minute and in commercial bleach (3% sodium hypochlorite) for 5 minutes, then finally rinsed six times with sterile water. Seeds were germinated on water agar plates at 30°C. Prior to inoculation, pots filled with 300 mL of air-dried vermiculite were autoclaved. Each pot was inoculated with a 100 mL soil sample, planted with 4 seedlings and received equal amounts of water. Each individual soil sample was divided into two pots: 3 populations * 3 soil samples/population * 4 plants/pot * 2 pots/soil sample = 72 plants. Plants received autoclaved water when necessary and were harvested after eight to twelve weeks of growth.

On average, three root nodules per individual plant were collected. The nodules were surface-sterilized in 70% ethanol for 1 minute and in commercial bleach (3% sodium hypochlorite) for 5 minutes, then finally rinsed six times with sterile water. Prior to the isolation of the rhizobia, root nodules were rolled over a peptone yeast extract agar plate containing nalidixic acid, $CaCl_2$ (7 mM) and $MgSO_4$ (10 mM) (*i.e.*, PYNal) to test the effectiveness of surface sterilization. The isolation of rhizobia was performed by squashing and streaking on PYNal agar plates. After an incubation period of three days at 30°C, all rhizobial isolates from successfully surface-sterilized nodules were purified by subculturing. The isolates were stored at -80°C in 50% glycerol for long-term storage.

Fig. S1. (A) Map of the Americas showing the WGS 84 coordinates of the sampled common bean fields given in latitude and longitude. The map was obtained from http://www.imagui.com/a/mapa-de-america-latina-sin-nombres-cdKbGxaoX.
(B) Pie charts showing which percentage of the sampled rhizobia and phages originate from which of the four common bean fields.

A

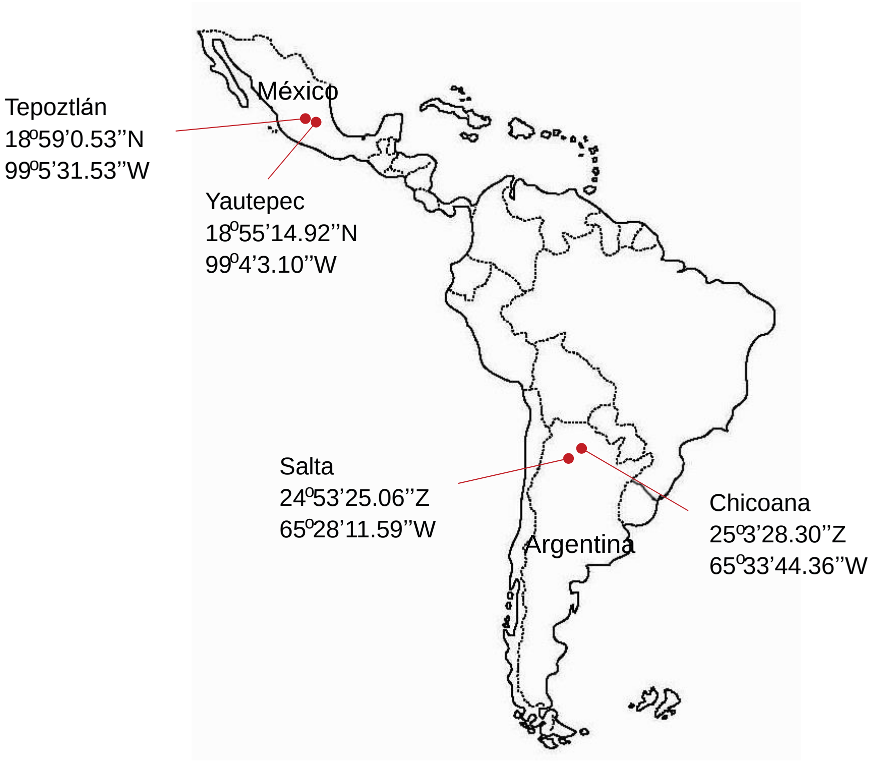

B

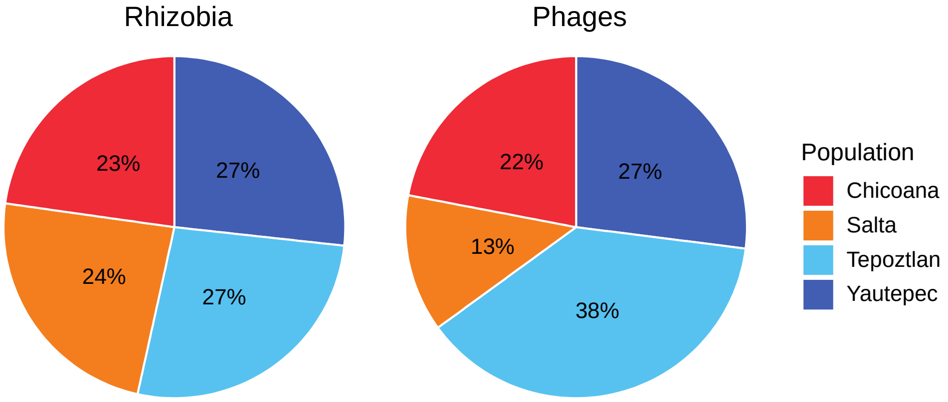

Fig. S2. Distribution of *Rhizobium* species (A) and phage families (B) across common bean fields.

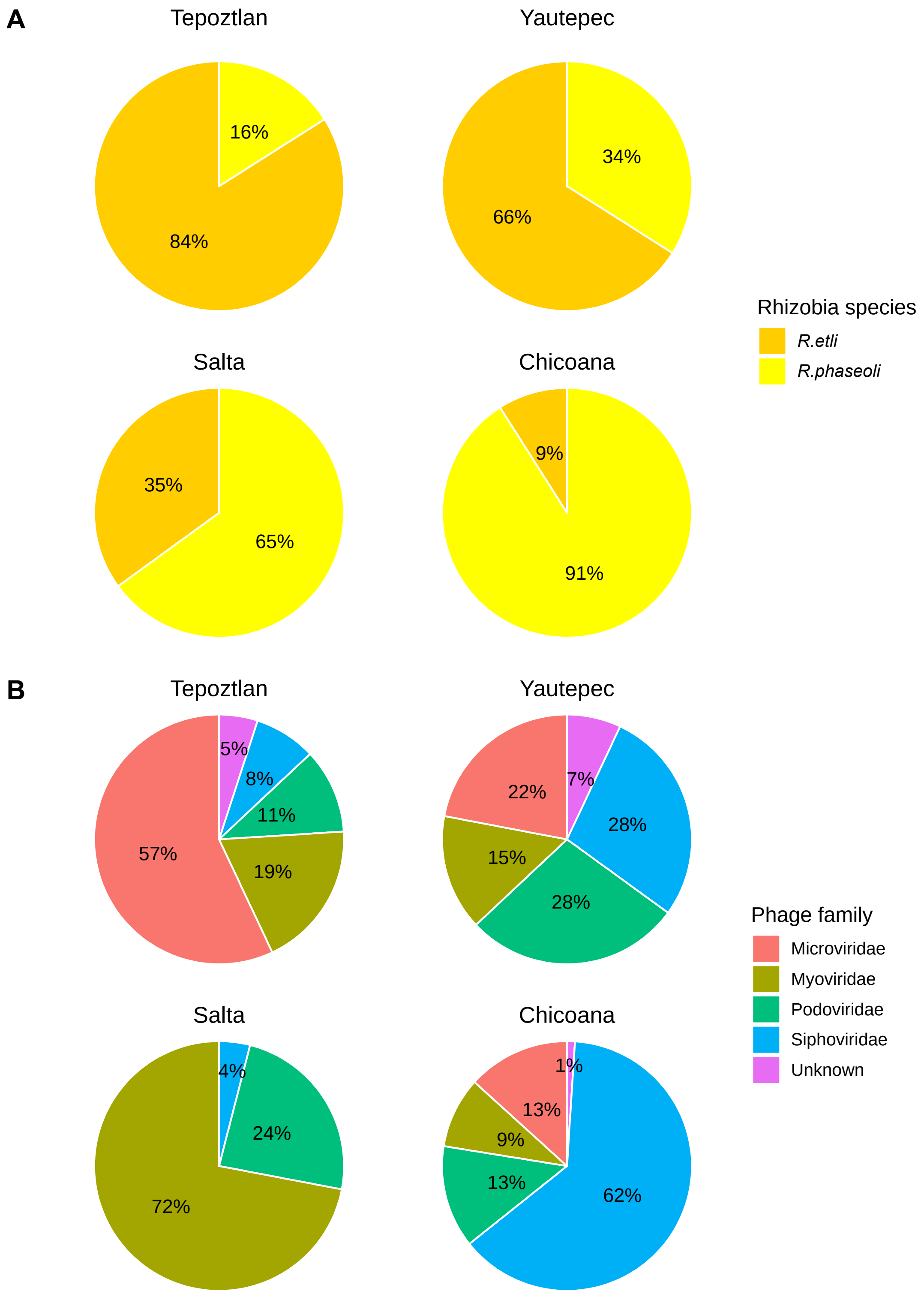

Fig S3. Heat map showing the products of ANIm (ANI by Mummer) and coverage. Phage families defined by ANIm and coverage. Genomes with a minimum of 80% nucleotide identity over an alignment covering at least 60% of the length of the smaller genome were grouped into a genomic family or phage genomic type (PGT; see Material and Methods).

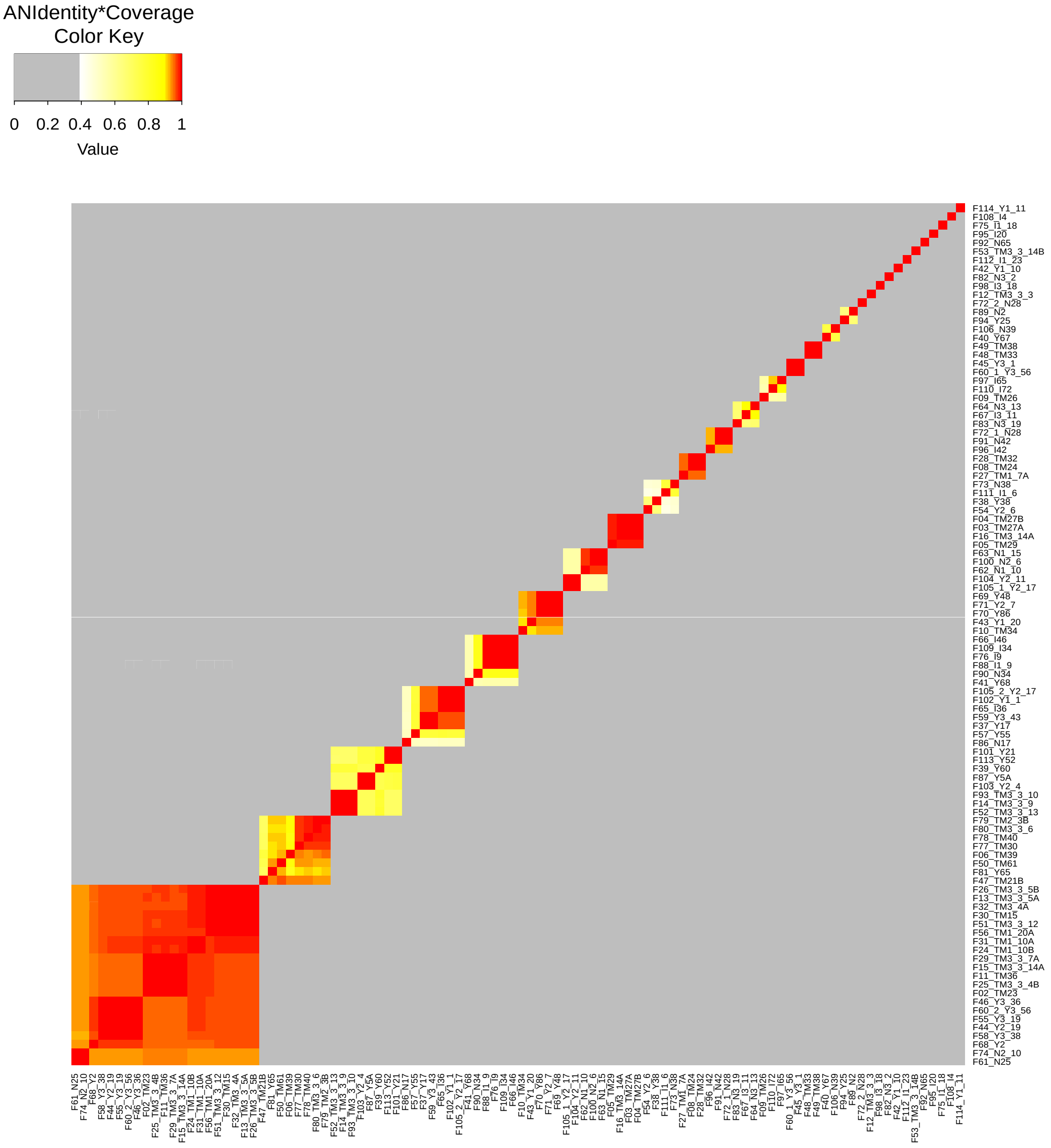

Table S1. List of SC strains with their taxonomic affiliation and the phages that were obtained using the respective strains.

Code	Name	Species and symbiovar identity	Origin	# isolated phages
1	N1314	*R. etli* sv. phaseoli	Tepoztlan, Morelos, Mexico	2
2	N741	*R. etli* sv. phaseoli	Tepoztlan, Morelos, Mexico	2
3	N561	*R. etli* sv. phaseoli	Tepoztlan, Morelos, Mexico	2
4	N771	*R. phaseoli* sv. phaseoli	Tepoztlan, Morelos, Mexico	2
5	N161	*R. phaseoli* sv. phaseoli	Tepoztlan, Morelos, Mexico	5
6	R72	*R. gallicum* sv. phaseoli	Tepoztlan, Morelos, Mexico	0
7	R634	*R. leguminosarum* sv. phaseoli	Tepoztlan, Morelos, Mexico	1
8	R744	*R. phaseoli* sv. phaseoli	Tepoztlan, Morelos, Mexico	1
9	R635	*R. leguminosarum* sv. phaseoli	Tepoztlan, Morelos, Mexico	2
10	N671	*R. phaseoli* sv. phaseoli	Tepoztlan, Morelos, Mexico	1
11	N261	*R. phaseoli* sv. phaseoli	Tepoztlan, Morelos, Mexico	1
12	R650	*R. phaseoli* sv. phaseoli	Tepoztlan, Morelos, Mexico	2
13	R723	*R. phaseoli* sv. phaseoli	Tepoztlan, Morelos, Mexico	1
14	R611	*R. phaseoli* sv. phaseoli	Tepoztlan, Morelos, Mexico	1
15	R620	*R. phaseoli* sv. phaseoli	Tepoztlan, Morelos, Mexico	2
16	R693	*R. gallicum* sv. phaseoli	Tepoztlan, Morelos, Mexico	1
17	R630	*R. phaseoli* sv. phaseoli	Tepoztlan, Morelos, Mexico	4
18	R711	*R. gallicum* sv. phaseoli	Tepoztlan, Morelos, Mexico	0
19	N122	*R. leguminosarum* sv. phaseoli	Tepoztlan, Morelos, Mexico	0
20	N324	*R. leguminosarum* sv. phaseoli	Tepoztlan, Morelos, Mexico	1
21	N841	*R. phaseoli* sv. phaseoli	Tepoztlan, Morelos, Mexico	1
22	N541	*R. leguminosarum* sv. phaseoli	Tepoztlan, Morelos, Mexico	1
23	N621	*R. etli* sv. phaseoli	Tepoztlan, Morelos, Mexico	2
24	N113	*R. etli* sv. phaseoli	Tepoztlan, Morelos, Mexico	1
25	N6212	*R. etli* sv. phaseoli	Tepoztlan, Morelos, Mexico	4
26	N4311	*R. leguminosarum* sv. phaseoli	Tepoztlan, Morelos, Mexico	1
27	N831	*R. phaseoli* sv. phaseoli	Tepoztlan, Morelos, Mexico	3
28	N731	*R. etli* sv. phaseoli	Tepoztlan, Morelos, Mexico	1
29	N931	*R. phaseoli* sv. phaseoli	Tepoztlan, Morelos, Mexico	1
30	N941	*R. leguminosarum* sv. phaseoli	Tepoztlan, Morelos, Mexico	2
31	N1341	*R. etli* sv. phaseoli	Tepoztlan, Morelos, Mexico	0
32	N871	*R. etli* sv. phaseoli	Tepoztlan, Morelos, Mexico	2
33	R339	*R. leguminosarum* sv. phaseoli	Tepoztlan, Morelos, Mexico	2
34	CFN42	*R. etli* sv. phaseoli	Guanajuato, Mexico	3
35	CIAT652	*R. phaseoli* sv. phaseoli	Costa Rica	1
36	GR56	*R. phaseoli* sv. phaseoli	Spain	2
37	KIM5	*R. phaseoli* sv. phaseoli	Idaho, USA	2
38	Bra5	*R. phaseoli* sv. phaseoli	Brazil	4
39	S20	*R. phaseoli* sv. phaseoli	Spain	2
40	CIAT894	*R. leguminosarum* sv. phaseoli	Colombia	2
41	Mim1	*R. etli* sv. mimosae	Huautla Morelos, Mexico	1
42	IE4771	*R. phaseoli* sv. giardinii	Puebla, Mexico	2
43	IE4803	*R. phaseoli* sv. phaseoli	Puebla, Mexico	1
44	IE4872	*R. gallicum* sv. gallicum	Puebla, Mexico	1
45	IE951	*R. phaseoli* sv. phaseoli	Puebla, Mexico	3
46	IE1004	*R. phaseoli* sv. phaseoli	Puebla, Mexico	3
47	IE1006	*R. phaseoli* sv. phaseoli	Puebla, Mexico	0
48	IE2737	*R. phaseoli* sv. phaseoli	Puebla, Mexico	1
49	IE4777	*R. phaseoli* sv. phaseoli	Puebla, Mexico	0
50	IE4810	*R. phaseoli* sv. phaseoli	Puebla, Mexico	0
51	IE6766	*R. gallicum* sv. phaseoli	Michoacan, Mexico	0
52	IE6794	*R. leguminosarum* sv. phaseoli	Michoacan, Mexico	1
53	IE6833	*R. etli* sv. phaseoli	Guanajuato, Mexico	0
54	IE6840	*R. etli* sv. phaseoli	Guanajuato, Mexico	0
55	IE6845	*R. etli* sv. phaseoli	Guanajuato, Mexico	1
56	IE6854	*R. etli* sv. phaseoli	Guanajuato, Mexico	0
57	IE6868	*R. etli* sv. phaseoli	Guanajuato, Mexico	0
58	IE6896	*R. phaseoli* sv. phaseoli	Puebla, Mexico	0
59	CNPAF512	*R. phaseoli* sv. phaseoli	Brazil	0
60	TAL182	*R. etli* sv. phaseoli	Hawaii, USA	1
61	NXC12	*R. etli* sv. mimosae	Huautla, Morelos, Mexico	1
62	NXC14	*R. etli* sv. mimosae	Huautla, Morelos, Mexico	0
63	MIM2	*R. phaseoli* sv. phaseoli	Huautla, Morelos, Mexico	0
64	MIM7-4	*R. etli* sv. mimosae	Huautla, Morelos, Mexico	0
65	INC 1-2	*R. phaseoli* sv. phaseoli	Guanajuato, Mexico	4
66	INC 2-5	*R. phaseoli* sv. phaseoli	Guanajuato, Mexico	1
67	TUX10P	*R. phaseoli* sv. phaseoli	Tuxtlas Veracruz, Mexico	1
68	TUX16P	*R. leguminosarum* sv. phaseoli	Tuxtlas Veracruz, Mexico	1
69	TUX1712m	*R. phaseoli* sv. phaseoli	Tuxtlas Veracruz, Mexico	0
70	TUX250P	*R. phaseoli* sv. phaseoli	Tuxtlas Veracruz, Mexico	0
71	CH24-10	*R. phaseoli* sv. phaseoli	Puebla, Mexico	0
72	343	*R. etli* sv. mimosae		1
73	F6	*R. etli* sv. phaseoli		0
74	B27	*R. phaseoli* sv. phaseoli		1
75	BAT-4	*R. etli* sv. phaseoli	Zacatecas, Mexico	0
76	BAT-49	*R. phaseoli* sv. phaseoli	Zacatecas, Mexico	0
77	BAT-32	*R. etli* sv. phaseoli	Zacatecas, Mexico	0
78	BAT-95	*R. phaseoli* sv. phaseoli	Zacatecas, Mexico	0
79	D5	*R. phaseoli* sv. phaseoli	Durango, Mexico	0
80	D11	*R. phaseoli* sv. phaseoli	Durango, Mexico	0
81	D13	*R. etli* sv. giardinii	Durango, Mexico	0
82	D21	*R. etli* sv. giardinii	Durango, Mexico	0
83	PS14	*R. phaseoli* sv. phaseoli		0
84	6C-1	*R. gallicum* sv. phaseoli	Spain	0
85	14C-1	*R. phaseoli* sv. phaseoli	Spain	0
86	14C-2	*R. phaseoli* sv. phaseoli	Spain	1
87	8NJ-2	*R. etli* sv. phaseoli	Spain	0
88	17NJ-2	*R. gallicum* sv. phaseoli	Spain	0
89	21NJ-2	*R. gallicum* sv. phaseoli	Spain	0
90	6PR-1	*R. phaseoli* sv. phaseoli	Spain	1
91	GR10	*R. gallicum* sv. phaseoli	Spain	0
92	GR14	*R. phaseoli* sv. phaseoli	Spain	0
93	GR62	*R. phaseoli* sv. phaseoli	Spain	0
94	GR75	*R. phaseoli* sv. phaseoli	Spain	0

Table S2. Primer sequences used for the partial amplification of the indicated *Rhizobium* and phage genes, for the taxonomic identification of rhizobia, and for the identification of phage genomic types. The following thermal cycling protocol was used: 94°C for 10 min, 30 X (94°C for 1 min, T_a for 1 min and 72°C for 1 min) and 72°C for 10 min. T_a is the annealing temperature: 57°C for rhizobium primers and 60°C for phage primers.

Rhizobium sp.	**Gene target/annotation**	**Direction**	**Sequence (5´-3´)**
Rhizobium sp.	*nodC*	Forward	GCTTTCAAGGGCAGTAGCAG
		Reverse	CCTCGGGAGTTCTGAAGATC
Rhizobium sp.	*dnaB*	Forward	CGCCGCTCGAAAGATTGCAG
		Reverse	TCATCATCGAGCAGGCGAC
Rhizobium sp.	*recA*	Forward	GACAAAAGCAAGGCACTTGAA
		Reverse	ACATCACGCCGATCTTCATGC
**Phage Genomic Type**			
F02	Hypothetical	Forward	CGTTCACGAAAACRCGCACY
		Reverse	TCGCATAGAACTKGTGGCGT
F03	Putative coat protein	Forward	CGATATCAACCTCGGCGTA
		Reverse	TTGTCGATGACCTTCCAGCC
F06	Phage tail sheath protein	Forward	AGGTGGTGCTGATGGTTCTG
		Reverse	TGGTGAATCTGCGACGAACA
F08	Putative major capsid protein	Forward	GCCGGTTCATGGCGTATTTC
		Reverse	GTGGCGTTCCAGTTGTTGAC
F10	Putative major capsid protein	Forward	CGATACCATCATCCTGGCCC
		Reverse	GATAGCGGCCTTGGGGATAC
F14	Terminase	Forward	GGCGAACGTGAACAAGTTCC
		Reverse	TGTTGAACCCGTCCAGATGG
F37	Putative terminase	Forward	CAAGATYAACCTGCTYGCGC
	protein large subunit	Reverse	GGTTTGAGCGCAATGATCCC
F38	Terminase subunit A	Forward	GAGCTTCCCGATACTTCCGG
		Reverse	AGCTGGACACGAGCAGTAAC
F40	Putative major capsid protein	Forward	TCTTCATCGATCCGGATGCG
		Reverse	GAAACCCGGCTCCTTGAAGA
F41	Tail completion and	Forward	GTCCACGAAACCATCTAACAACG
	sheath stabilizer protein	Reverse	GACGTCGACTTCCACGTTCT
F45	putative hydrolase	Forward	TATCGGGATCCCCTACGTCC
		Reverse	GGAGCCGATGAACACTCCAA
F62	Putative tail assembly	Forward	CGACGTCGACTTCTACGAGG
	protein	Reverse	GATCATGCCCGGTTCGATCT
F64	Major capsid protein	Forward	CAGTTCCGCAAGACCTCTGT
		Reverse	CCAGTTGACATGGGACTGCT
F75	DNA polymerase	Forward	TCATCGATCTCACACGCTCG
		Reverse	GGTCTTGCGGCTTGTTGAAG

Table S3. Groups of the phage host range defined as phage phenotype groups (PPGs), and groups of *Rhizobium* susceptibility expressed as *Rhizobium* phenotype groups (RPGs). The minimum and maximum number of infections, phage genomic types (PGT) per PPG, and *Rhizobium* STs per RPG are given.

Phage Phenotypic Groups (PPGs)					*Rhizobium* Phenotypic Groups (RPGs)				
	Phages	# min infections	# max infections	PGT´s (F´s)		Strains	# min infections	# max infections	ST´s
PPG-1	42	97	132	2 & 8	RPG-1	40	42	55	3, 5 & 41
PPG-2	24	175	214	62 & 108	RPG-2	38	105	142	5, 7, 10, 20, 26, 36, 39
PPG-3	9	173	210	6, 10, 14 & 62	RPG-3	36	87	145	21, 22, 33 & 34
PPG-4	7	71	94	41	RPG-4	19	48	75	14, 15, 16 & 17
PPG-5	6	50	69	2, 14, 37 & 114	RPG-5	12	56	63	18
PPG-6	5	90	113	38	RPG-6	9	93	137	5, 11, 27, 30 & 36
PPG-7	4	73	90	3	RPG-7	7	68	88	22, 27 & 34
PPG-8	4	52	58	14	RPG-8	5	84	122	22 & 32
PPG-9	4	63	87	2	RPG-9	5	58	68	23 & 35
PPG-10	4	89	124	6, 37 & 62	RPG-10	4	36	60	22, 33 & 34
PPG-11	3	120	121	2 & 9	RPG-11	4	50	69	13, 15 & 40
PPG-12	3	107	116	38 & 40	RPG-12	4	64	86	22 & 34
PPG-13	3	41	42	45	RPG-13	4	62	96	6 & 9
PPG-14	3	136	151	6	RPG-14	3	62	64	25
PPG-15	3	76	88	2	RPG-15	2	82	85	31
PPG-16	2	151	152	62	RPG-16	2	94	101	39
PPG-17	2	51	56	41	RPG-17	2	104	114	8 & 28
PPG-18	2	83	87	2	RPG-18	2	80	83	37
PPG-19	2	9	12	2	RPG-19	2	82	101	5 & 10
PPG-20	2	113	120	10	Singleton RPGs	29	20	129	1, 2, 4, 5, 10, 11, 12, 19, 22, 23, 24, 29, 33, 34, 35, 36, 37, 38
PPG-21	2	62	66	14					
PPG-22	2	47	53	14					
PPG-23	2	67	67	64					
PPG-24	2	18	25	75					
Singleton PPGs	57	5	163	2, 3, 6, 9, 10, 12, 14, 37, 38, 40, 41, 42, 48, 53, 62, 64, 72_1, 72_2, 75, 82, 89, 92, 95, 98, 112					

Table S4. Relative abundance of chromosomal sequence types (ST) among common bean fields and regions given in percentages (rounded to two decimals).

		Mexico		Argentina			
**ST code**	**Species**	**Tepoztlán**	**Yautepec**	**Salta**	**Chicoana**	**Mexico**	**Argentina**
		**n=61**	**n=61**	**n=53**	**n=54**	**n=122**	**n=107**
1	*R. phaseoli*	0.00	0.00	1.85	0.00	0.00	0.93
2	*R. phaseoli*	0.00	0.00	0.00	1.89	0.00	0.93
3	*R. phaseoli*	1.64	0.00	0.00	0.00	0.82	0.00
4	*R. phaseoli*	1.64	0.00	0.00	0.00	0.82	0.00
5	*R. phaseoli*	1.64	29.51	18.52	83.00	15.57	50.47
6	*R. phaseoli*	0.00	0.00	1.85	0.00	0.00	0.93
7	*R. phaseoli*	0.00	0.00	1.85	0.00	0.00	0.93
8	*R. etli*	0.00	0.00	1.85	0.00	0.00	0.93
9	*R. phaseoli*	0.00	0.00	0.00	5.66	0.00	2.80
10	*R. phaseoli*	8.20	1.64	0.00	0.00	4.92	0.00
11	*R. phaseoli*	1.64	3.28	0.00	0.00	2.46	0.00
12	*R. phaseoli*	1.64	0.00	0.00	0.00	0.82	0.00
13	*R. phaseoli*	0.00	0.00	3.70	0.00	0.00	1.87
14	*R. phaseoli*	0.00	0.00	1.85	0.00	0.00	0.93
15	*R. phaseoli*	0.00	0.00	3.70	0.00	0.00	1.87
16	*R. phaseoli*	0.00	0.00	29.63	0.00	0.00	14.95
17	*R. phaseoli*	0.00	0.00	1.85	0.00	0.00	0.93
18	*R. etli*	0.00	0.00	22.22	0.00	0.00	11.21
19	*R. etli*	0.00	0.00	1.85	0.00	0.00	0.93
20	*R. etli*	0.00	0.00	0.00	1.89	0.00	0.93
21	*R. etli*	1.64	0.00	0.00	0.00	0.82	0.00
22	*R. etli*	22.95	8.20	0.00	0.00	15.57	0.00
23	*R. etli*	0.00	6.56	0.00	0.00	3.28	0.00
24	*R. etli*	1.64	1.64	0.00	0.00	1.64	0.00
25	*R. etli*	0.00	0.00	0.00	5.66	0.00	2.80
26	*R. etli*	0.00	1.64	0.00	0.00	0.82	0.00
27	*R. etli*	0.00	3.28	0.00	0.00	1.64	0.00
28	*R. etli*	0.00	0.00	1.85	0.00	0.00	0.93
29	*R. etli*	0.00	1.64	0.00	0.00	0.82	0.00
30	*R. etli*	0.00	4.92	0.00	0.00	2.46	0.00
31	*R. etli*	0.00	3.28	0.00	0.00	1.64	0.00
32	*R. etli*	6.56	0.00	0.00	0.00	3.28	0.00
33	*R. etli*	4.92	1.64	0.00	0.00	3.28	0.00
34	*R. etli*	45.90	11.48	0.00	0.00	28.69	0.00
35	*R. etli*	0.00	8.20	0.00	0.00	4.10	0.00
36	*R. etli*	0.00	6.56	0.00	0.00	3.28	0.00
37	*R. etli*	0.00	4.92	0.00	0.00	2.46	0.00
38	*R. etli*	0.00	1.64	0.00	0.00	0.82	0.00
39	*R. etli*	0.00	0.00	5.56	0.00	0.00	2.80
40	*R. etli*	0.00	0.00	1.85	0.00	0.00	0.93
41	*R. etli*	0.00	0.00	0.00	1.89	0.00	0.93

Table S5. Degrees of freedom (df), pseudo-*F*-values and *p*-values of PERMANOVA analyses testing the significance of compositional differences of *Rhizobium* chromosomal STs and phage PGTs across all four common bean fields and both common bean gene pools based on four distance matrices: Bray-Curtis dissimilarity matrix and Jaccard dissimilarity matrix. P-values for fitting the PCNM values on the PCoA are also given. Significant ($p < 0.05$) values are indicated in bold.

Dataset	Distance matrix	Among fields			Among gene pools			PCNM fit
		df	Pseudo -*F*-value	*p*-value	*df*	Pseudo -*F*-value	*p*-value	*p*-value
Rhizobium chromosomal STs	Bray-Curtis	3,8	5.0	**0.001**	1,10	5.0	**0.005**	**0.002**
	Jaccard	3,8	3.6	**0.001**	1,10	3.4	**0.005**	**0.001**
R. phaseoli chromosomal STs	Bray-Curtis	3,8	2.4	**0.009**	1,10	2.0	0.070	0.116
	Jaccard	3,8	2.2	**0.013**	1,10	1.8	0.063	**0.021**
R. etli chromosomal STs	Bray-Curtis	3,6	4.1	**0.009**	1,8	4.0	**0.001**	**0.002**
	Jaccard	3,6	2.9	**0.006**	1,8	2.9	**0.002**	**0.003**
Phage genomic types	Bray-Curtis	3,10	3.3	**0.001**	1,12	3.4	**0.001**	**0.001**
	Jaccard	3,10	2.5	**0.001**	1,12	2.7	**0.001**	**0.030**

Table S6. Features and accession numbers of the obtained phage genomes.

(See spreadsheet available in online supplementary materials)

Table S7. Relative abundance of phage genomic types among common bean fields and regions given in percentages (rounded to two decimals). Taxonomic family identities are given.

PGT	Taxonomic family	Mexico Tepoztlán n=74	Mexico Yautepec n=50	Argentina Salta n=45	Argentina Chicoana n=26	Mexico n=124	Argentina n=71
F02	Microviridae	48.65	22.00	0.00	11.11	37.90	7.04
F03	Podoviridae	6.76	0.00	0.00	0.00	4.03	0.00
F06	Myoviridae	18.92	2.00	0.00	0.00	12.10	0.00
F08	Microviridae	12.16	0.00	0.00	0.00	7.26	0.00
F09	Myoviridae	1.35	0.00	7.69	0.00	0.81	2.82
F10	Podoviridae	1.35	12.00	0.00	0.00	5.65	0.00
F12	Siphoviridae	1.35	0.00	0.00	0.00	0.81	0.00
F14	Siphoviridae	5.41	16.00	0.00	0.00	9.68	0.00
F37	Myoviridae	0.00	12.00	3.85	2.22	4.84	2.82
F38	Podoviridae	0.00	12.00	3.85	2.22	4.84	2.82
F40	Microviridae	0.00	2.00	0.00	2.22	0.81	1.41
F41	Myoviridae	0.00	4.00	42.31	2.22	1.61	16.90
F42	Podoviridae	0.00	2.00	0.00	0.00	0.81	0.00
F45	Siphoviridae	0.00	8.00	0.00	0.00	3.23	0.00
F48	Podoviridae	2.70	0.00	3.85	2.22	1.61	2.82
F53	Siphoviridae	1.35	0.00	0.00	0.00	0.81	0.00
F62	Siphoviridae	0.00	4.00	0.00	60.00	1.61	38.03
F64	Podoviridae	0.00	0.00	7.69	4.44	0.00	5.63
F72_1	Myoviridae	0.00	0.00	3.85	4.44	0.00	4.23
F72_2	Siphoviridae	0.00	0.00	0.00	2.22	0.00	1.41
F75	Myoviridae	0.00	0.00	11.54	0.00	0.00	4.23
F82	Podoviridae	0.00	0.00	0.00	2.22	0.00	1.41
F89	Podoviridae	0.00	2.00	0.00	2.22	0.81	1.41
F92	Podoviridae	0.00	0.00	0.00	2.22	0.00	1.41
F95	Podoviridae	0.00	0.00	3.85	0.00	0.00	1.41
F98	Podoviridae	0.00	0.00	3.85	0.00	0.00	1.41
F108	Siphoviridae	0.00	0.00	3.85	0.00	0.00	1.41
F112	Podoviridae	0.00	0.00	3.85	0.00	0.00	1.41
F114	Siphoviridae	0.00	2.00	0.00	0.00	0.81	0.00

Table S8. Mantel's r statistics and P-values for correlations among compositional differences of *Rhizobium* chromosomal STs, PGTs, RPGs, and PPGs across all four common bean fields. Correlations using the Jaccard distance matrix (above the diagonal, in gray), and the Bray-Curtis distance matrix (below the diagonal).

	**Data set**			
**Data set**	**STs**	**PGTs**	**RPGs**	**PPG**
**STs**	-	$r = 0.54$; $p = 0.003$	$r = 0.68$; $p = 0.001$	$r = 0.68$; $p = 0.002$
**PGTs**	$r = 0.43$; $p = 0.002$	-	$r = 0.40$; $p = 0.014$	$r = 0.63$; $p = 0.001$
**RPGs**	$r = 0.87$; $p = 0.001$	$r = 0.44$; $p = 0.009$	-	$r = 0.52$; $p = 0.004$
**PPGs**	$r = 0.46$; $p = 0.007$	$r = 0.76$; $p = 0.001$	$r = 0.47$; $p = 0.010$	-
